# Implementation of digital home monitoring and management of respiratory disease

**DOI:** 10.1097/MCP.0000000000000965

**Published:** 2023-05-02

**Authors:** Hilary Pinnock, Chi Yan Hui, Job F.M. van Boven

**Affiliations:** a Usher Institute, The University of Edinburgh, UK; b Department of Clinical Pharmacy and Pharmacology, Groningen Research Institute for Asthma and COPD (GRIAC), University Medical Center Groningen, University of Groningen, The Netherlands

**Keywords:** digital inequalities, digital technology, electronic monitoring, medical device regulation, respiratory care

## Abstract

Digital respiratory monitoring interventions (e.g. smart inhalers and digital spirometers) can improve clinical outcomes and/or organizational efficiency, and the focus is shifting to sustainable implementation as an approach to delivering respiratory care. This review considers key aspects of the technology infrastructure, discusses the regulatory, financial and policy context that influence implementation, and highlights the over-arching societal themes of equity, trust and communication.

**Recent findings:**

Technological requirements include developing interoperable and connected systems; establishing stable, wide internet coverage; addressing data accuracy and monitoring adherence; realising the potential of artificial intelligence; and avoiding clinician data overload. Policy challenges include concerns about quality assurance and increasingly complex regulatory systems. Financial barriers include lack of clarity over cost-effectiveness, budget impact and reimbursement. Societal concerns focus on the potential to increase inequities because of poor e-health literacy, deprivation or lack of available infrastructure, the need to understand the implications for patient/professional interactions of shifting care to remote delivery and ensuring confidentiality of personal data.

**Summary:**

Understanding and addressing the implementation challenges posed by gaps in policy, regulatory, financial, and technical infrastructure is essential to support delivery of equitable respiratory care that is acceptable to patients and professionals.

## INTRODUCTION

E-Health technology is widely promoted as contributing to accessible [1], efficient [2] and patient-centred healthcare [3]. Within respiratory care, digital home monitoring devices, such as smart inhalers and digital spirometers enable clinicians to monitor real-time progress of disease [4], tailor care [5,6], initiate and monitor treatment [7], undertake informed remote consultations [8], promote inhaler technique [9], medication adherence [10^▪^] and support self-management [11]. The data generated by digital home monitoring can be used to target resources and monitor health events [12]. 

**Box 1:**
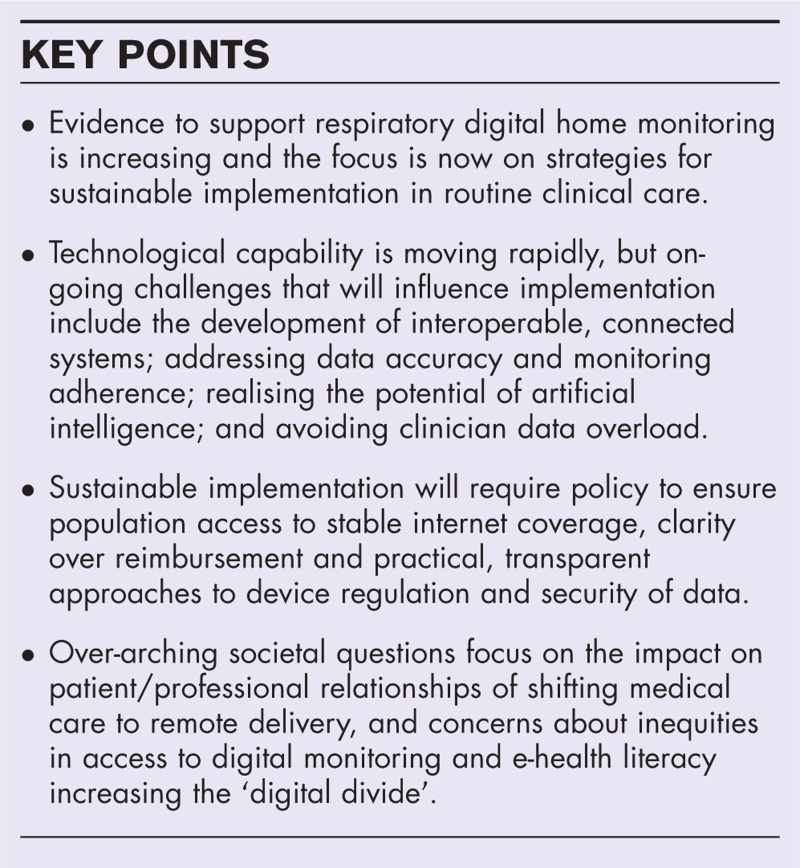
no caption available

## IMPLEMENTATION STRATEGIES

As evidence accumulates of effectiveness of disease or task-specific digital interventions on clinical outcomes and/or organizational efficiency [10^▪^,^13^–^18^,19^▪^,^20^], the focus is shifting to broader strategies that could promote implementation as a sustainable approach to delivering respiratory care [21]. However, successful implementation is complex requiring understanding of the patient, professional and organizational perspectives [22], as well as developing the technology infrastructure [23], working within the political, economic and regulatory policy context [24^▪^], and addressing societal concerns about health literacy, exacerbating digital inequalities [25], the impact of remote care on patient/professional communication [26], engendering trust and ensuring data security.

## THE FOCUS OF THIS REVIEW

Within the broad range of digital interventions, in this review we focus on home monitoring of respiratory disease and remote delivery of care. We will provide an overview of available technologies and consider key aspects of the technology infrastructure, regulatory, financial and policy context that have a major influence on the success of implementing digital respiratory care within routine clinical care. Finally, we will highlight some of the over-arching themes of equity, trust and communication that will influence societal acceptance of home monitoring and remote care.

## DEVELOPING DIGITAL HOME MONITORING TECHNOLOGY AND INFRASTRUCTURE

Table 1 summarizes some existing digital home monitoring interventions in a number of respiratory conditions that have benefit in supporting patients to manage their conditions, reducing hospitalization, and facilitating remote consultation [10^▪^,^13^,^18^,^27^–^59^]. These interventions have proved to be feasible and effective in research contexts and potentially could be developed for implementation in routine practice. Technological challenges that will need to be overcome include: developing interoperable and connected systems; establishing stable wide internet coverage; addressing data accuracy and adherence to monitoring; realizing the potential of artificial intelligence and virtual reality and avoiding clinician data overload.

**Table 1:** Summary table of exemplar digital monitoring in respiratory disease

Clinical work	Current norm/standard of care/promising interventions (clinician-led care)	What could be implemented in the future? (AI-enabled care)
Self-management with action plan advice	Use of retrospective datasets to Build rule-based algorithms to phenotype and categorize severity of the condition; Predict early exacerbations (e.g. in cystic fibrosis [27], COPD [28,29], asthma [30]); Provide advice to patients according to an action plan agreed with their clinician Prompt patients to explore online supportive information [31].	Connecting multiple smart devices, collecting real-time patient data, environmental data and retrospective datasets (e.g. EHR) from healthcare organisations to provide timely, personalized self-management advice to prevent exacerbations. AI could tailor information to the individual's condition, requirements, interests and context.
Clinician monitoring	Clinicians monitor home spirometry measurements to: Inform remote consultations for vulnerable patients (e.g. with ILD during the COVID-19 pandemic [32]) Follow-up after lung cancer surgery [33]	AI could monitor incoming patient data and flag alarms to clinicians to provide timely care to patients.
Remote monitoring to support diagnosis	Using retrospective EHR, patient self-reported data sets and CDSS to categorize suspected and confirmed COPD/asthma based on existing clinical guidelines [34–36].	AI could use real-time patient monitoring data to support clinician diagnosis, confirm disease status, or to identify environmental triggers
Stratification	Using retrospective EHR/patient-reported data sets, rule-based algorithms to stratify risk [30,37,38], Identify patients safe for discharge from ED; Initiate readmission prevention [39].	AI could collect on-going patient home monitoring data to identify high-risk patients, supporting clinicians to target early treatments, support and management strategies
Home monitoring to support remote consultation	Video consultation;[40] Follow-up chat by text messenger/telephone; Text reminder for consultation; E-mail or text home monitoring measurements to clinicians. (e.g. COPD [18], cystic fibrosis [41], asthma and other conditions [42])	AI interpretation of lung sounds, using a digital stethoscope for remote auscultation [43,44]. Online portal to view self-monitoring measurements to support assessment
Home monitoring rehabilitation and physical exercise	Online supervision of COPD rehabilitation [13], including a virtual therapist [45]. Using a game console as exercise treatment to patients with cystic fibrosis [46]. Remote exercise testing [47].	Future potential includes: eXtended reality, augmented reality, virtual reality, and mixed reality (xR, AR, VR, MR) physical trainings [48,49].
Medication adherence	Smart inhalers, app reminders, text reminders [10^▪^]. Video/mobile direct observation of therapy in TB, children with asthma, elderly in COPD [50].	Smart inhalers (asthma/COPD) or smart pill boxes (TB) to detect medication adherence and provide targeted intervention on device or via intelligent home assistance
Monitoring inhaler technique	Inhaler technique checking via video by nurses [51]. Inhaler technique training game for children with asthma [52].	Self-checking AI-mediated smart inhaler technique, augmented reality (AR), smartphone camera and microphone, and smart inhaler [53–55]
Psychological support	Monitoring the mental health conditions of the COPD patients and provide digital support and education [56].	Passive monitoring of patients’ behaviours (e.g. physical activity, geolocation, phone unlock duration, speech frequency and duration) to support clinicians to customize psychological therapy [57].
Lay-led monitoring	Patients with similar conditions share logs, asthma experience and opinion on treatment in the social forum [58].	3D virtual worlds such as the Metaverse for respiratory community, allowing new immersive sharing experience (e.g. virtual social event and workshop)
Monitoring environmental triggers at home	Generic tips to avoid indoor triggers available online (e.g. open windows, avoid aerosols and sprays, aerosols and sprays) [59]	AI could identify possible triggers relevant to the individual and provide targeted advice.

AI, artificial intelligence; CDSS, clinical decision support systems; COPD, chronic obstructive pulmonary disease; ED, emergency department; EHR, electronic health record; ILD, interstitial lung disease.

### Developing interoperable ‘connected’ systems

Monitoring a combination of disease markers is often helpful to assess control in, for example, asthma [30]. This requires collecting data from multiple devices, most of which only feedback data via their own branded app so that patients have to download multiple apps to their phones, and clinicians receive multiple health reports in different formats. Linking multiple devices and summarizing data in one common platform for patients/professionals will be an important practical advance, but work on a device-agnostic connected platform is mostly still in the pilot stages [60,61]. Further development is required to ensure the connection process is as simple as ‘plug and play’ to encourage adoption [62].

**FIGURE 1:**
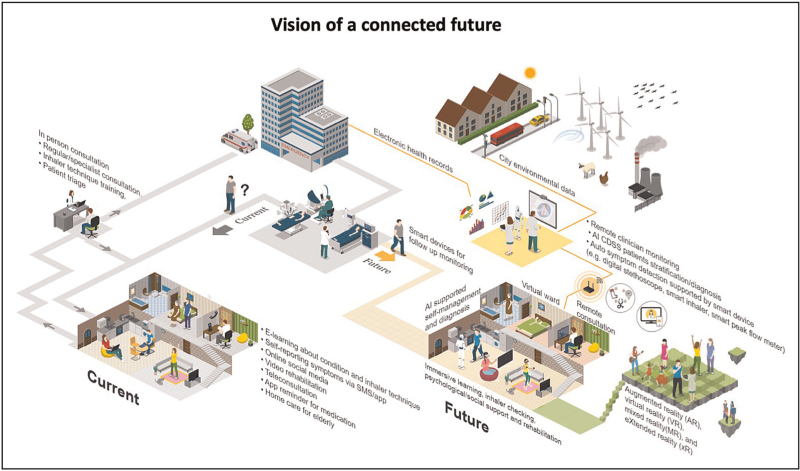
Vision of a connected future. On the left is the traditional referral pathway as the patient attends a clinic, may be referred to a hospital for tests or treatment and discharged home. Promising digital innovations exist (tele-consultations, on-line information, social fora, apps and reminders) are available but typically stand alone. Primary care and hospital use electronic health records (EHRs) but interoperability with different sectors or with patients is limited/nonexistent. On the right is a vision of an interconnected system in which artificial intelligence (AI)-supported self-management advice and clinical decision support is informed by a fully interoperable system. AI can use data from all these sources to tailor information provision and health promotion, monitor disease and environmental status and alert to increased risk, detect attacks and advise self-management actions, deliver home-based treatments (such as rehabilitation, hospital at home, psychological treatments) and provide social support and monitoring. Acknowledgement: the figure is created in the ICOGRAMS.

Most healthcare organizations use proprietary platforms, which are challenging to convert to a standardized format [e.g. using Fast Healthcare Interoperability Resources (FHIR)], to enable data sharing with other practices and hospitals. Practical barriers include lack of staff time and resources to map existing patient records to the new data format, and poor training to encourage adoption [23]. Customized solutions may allow data to move between local general practitioner practices, pharmacies and hospitals as the foundation to a future open data flow to all healthcare systems. Figure 1 illustrates a vision of a connected future.

### Establishing stable, wide internet coverage

A widely available stable internet connection is important for home monitoring that needs a timely action from clinicians (e.g. a virtual ward). If data transmission is interrupted, some devices can store measurements locally and resend them once connection is re-established, but others will lose any unsent measurements. To avoid this, patients are advised to conduct measurements in an area where they have a strong home wi-fi or mobile data signal. This can be a barrier in areas with low-resource availability, poor internet infrastructures or congestion because of high local demand [63^▪^]. Where signals are adequate, mobile data plans may be a backup solution for transferring patient data.

### Addressing data accuracy and adherence to monitoring

Incomplete datasets pose a challenge for artificial intelligence, which seeks to provide an accurate decision based on the assumption that the reported data reflect the truth [30]. In reality, patient self-reporting of symptoms carries an inherent risk of bias. Patients forget to record measurements, and some may manipulate data to complete missing logs. Missing data are not random: patients may stop logging when their condition is stable – or conversely when they feel too ill to submit readings [64,65]. Conversely, artificial intelligence can help to improve the quality of data by automatically reminding patients about missed logs to improve adherence to monitoring, though unmotivated patients typically ignore reminders or turn them off [66]. Reports of logging can enable clinicians to discuss nonadherence with the patient [30,67]. Artificial intelligence-enabled monitoring processes (for example, implemented on a smart spirometer) can quality check readings, provide feedback and coach the patient to improve accuracy of measurements [68^▪^]. Passive sensing can be used to collect patient data automatically and silently, which can reduce the human error in logging but privacy concerns may dissuade patients from adopting such systems [69].

### Realizing the potential of artificial intelligence and virtual reality

Artificial intelligence algorithms that use retrospective patient data and clinical guidelines to predict exacerbations or identify at-risk patients are increasingly common; however, these models represent populations, not individuals. Adaptive artificial intelligence can build personalized support by interacting regularly with patients and learning what the data mean to individuals. This process should be validated by clinicians.

Accelerated by the COVID-19 pandemic, augmented reality, virtual reality, mixed reality and eXtended reality can support home-based rehabilitation, checking inhaler technique, providing psychological and social support [48,49]. Patients can enter a 3D immersive environment to interact with clinicians; however, they would either need a smart phone/TV with a built-in camera and motion sensors, or purchase additional devices (such as virtual reality headset). The naked-eye 3D technology is an option to provide an immersive experience without a headset but some patients with visual disabilities and elderly will have difficulties in adopting such technologies.

Although artificial intelligence can enhance the home monitoring support to patients, there are deployment challenges in real-life clinical practice. Critically, liability for inappropriate decisions needs to be defined: the manufacturer of the device which may have generated an inaccurate reading; the developer of the artificial intelligence that may have provided a biased prediction based on limited historical data or generated in single ethnic group study; the healthcare provider who recommended the device in an inappropriate clinical situation; or the patient who may have misused the device or misinterpreted the automated advice. There is a need to build trust in the artificial intelligence so that it is perceived to be reliable, easy to use and secure by patients and clinicians to enhance adoption and the sustained use of the system [70–72].

### Avoiding clinician data overload

Clinicians want relevant home monitoring data, but do not want to be overloaded with a mass of irrelevant data [73]. This will be context-specific. For supporting self-management, clinicians need to decide on a core set of data and how much they need to make an accurate assessment. Where timely action is needed (e.g. monitoring hospital-at-home), continuous monitoring of multiple parameters is required and a dedicated team will need to be allocated time to monitor the data and intervene if needed, potentially supported by validated artificial intelligence to flag alarms.

## CHALLENGES OF THE POLICY, REGULATORY AND FINANCIAL CONTEXT

There are many respiratory apps and monitoring devices (e.g. digital spirometers, smart inhalers), as well as widely available technologies used for purposes (e.g. capturing lung sounds with the built-in microphone on a smart phone [74]) raising concerns about quality assurance. However effective, a novel home monitoring technology (e.g. in COPD [75], tuberculosis [76] and asthma [77]) will not be implemented in routine respiratory care if the efficacy–safety balance is not approved by regulatory bodies and the use appropriately resourced [68^▪^].

### Medical device regulation

In Europe, devices and apps fall under the Medical Device Regulation (MDR) where ‘monitoring’ is one of the possible goals of a device used for medical purposes. Devices used in clinical practice or placed on the market, require, for example, a European Conformity (CE) mark [78], Food and Drug Administration (FDA) approval [79] or other national equivalent [80]. In Europe, the manufacturer is responsible for identifying applicable requirements (checking which directive/harmonized standards apply; whether independent assessment by a notified body is needed), testing and documenting technical performance, and drafting the EU declaration of conformity of their device or application. There are several anomalies and pitfalls within this process:

#### Limitations of European Conformity marking

CE marking typically only certifies the manufacturer's assessment of health and safety requirements, and (depending on the class of device) not necessarily clinical effectiveness, user acceptability or health economic value all of which are important for successful implementation. There are calls for improved transparency in this process to enable healthcare systems to assess and judge the performance of technology [81], including databases listing key performance indicators that benchmark the technologies to help clinicians and patients to choose appropriate technology [82].

#### Drug–device combinations

**FIGURE 2:**
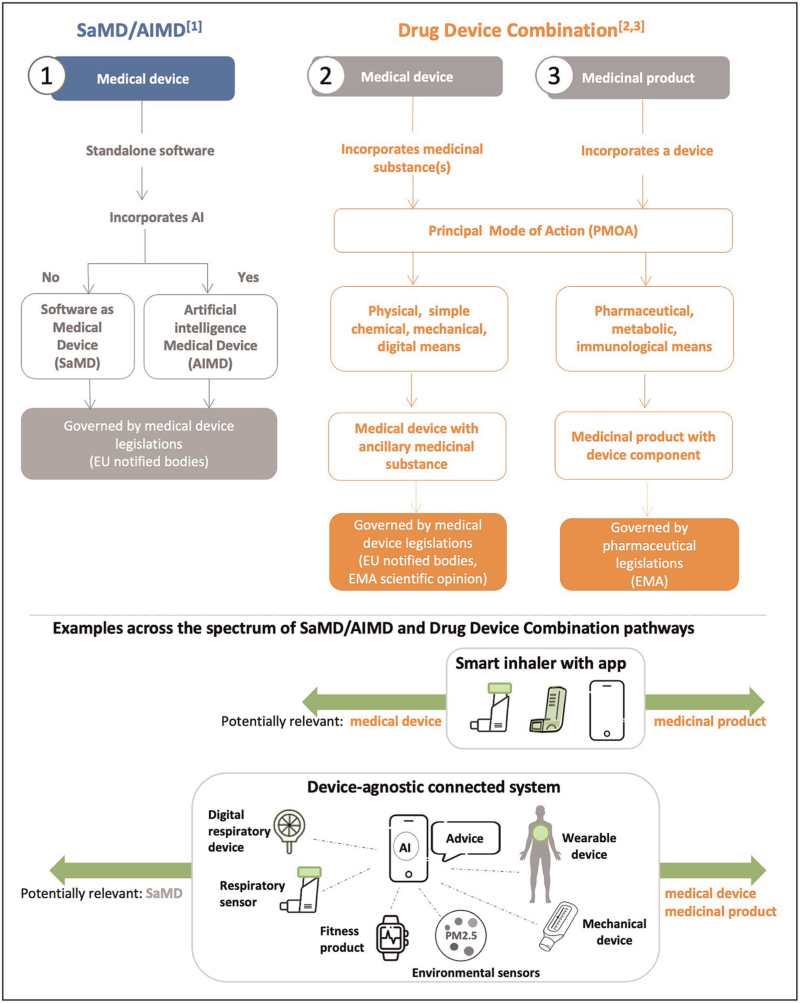
Medical device and medicinal product legislation pathways. Relevant regulation. 1. International medical device regulators forum, medical device updated includs SaMD and AIaMD, 2022, https://www.imdrf.org/meetings/web-conference-hosted-australia-0. 2. EMA. Medical Devices 2022 https://www.ema.europa.eu/en/human-regulatory/overview/medical-devices and updated for combined medicinal product and medical devices development: 2018 https://www.youtube.com/watch?v=fuewJ7gNXVI&t=403 s. 3. Reis ME, Bettencourt A, Ribeiro HM. The regulatory challenges of innovative customized combination products. *Front Med* 2022; 9 : 821094.

Although the MDR is relatively clear; challenges arise when technologies for home monitoring span different regulations (see Fig. 2). A respiratory example is smart inhalers that can remotely monitor medication adherence and inhaler technique [67,83,84^▪^]. These are ‘drug–device combinations’ as they contain a medical device, a software application as well as an active pharmaceutical ingredient so that both MDR and the European Medicines Agency (EMA) are relevant in regulatory assessment. An additional complication is that, although some device-specific software is included in the MDR assessment, more complex monitoring apps with an algorithm-based artificial intelligence might require approval as an Artificial Intelligence Medical Device (AIMD) [85].

In the United States, the FDA document ‘Digital Health Technologies for Remote Data Acquisition in Clinical Investigations’ [86], provides guidance on what is needed regarding selection, description, validation, usability and clinical endpoints when submitting digital (remote monitoring) technologies for approval. Examples include sensor-based hardware, software or combinations of multiple digital health technologies. Similar guidance for pathways is needed in the EU.

#### Software as medical device

Software applications are typically designed to support a single device – or linked devices from a single company; the registration process is currently unclear on how it applies to systems level connected platforms processing input from multiple sources. Clarification will be needed to enhance implementation towards an interoperable, device-agnostic connected system [60–62].

### Financial considerations

Although regulatory aspects are relatively uniform and well described across Europe and the United States [86], financial aspects, such as reimbursement, are still largely managed at a country, state or even health plan level. Within clinical studies, home monitoring devices are usually funded by study budgets, but in daily clinical practice, payment needs to come from elsewhere. This is a major barrier to implementation of home monitoring technologies and is one of the reasons that development often stalls at pilot/evaluation stage [87^▪^]. Any implementation strategy will need to address who will pay, for what and when.

In principle, a manufacturer could choose to sell its home monitoring device directly to the patient/consumer, but this will be unaffordable for many patients raising concerns about inequity. Manufacturers, therefore, usually aim for reimbursement of their device by health insurers. In many countries, this requires a solid business case, including evidence of cost-effectiveness [88] and reasonable budget impact [89]. A challenge here is that the socio-economic context, as well as health economic guidelines and required dossiers are often country-specific [89], and it is time-consuming and expensive to compile the right data for each individual country before gaining global market access – a particular challenge for small technology companies. Consequently, availability and reimbursement of home monitoring can show large inter-country differences.

Moreover, even within individual countries, reimbursement pathways for digital home monitoring devices are still in their infancy as devices/applications/digital systems do not always fit within traditional reimbursement silos or schemes. For example, a novel smart inhaler with an established pharmacologic compound might be judged – and priced – as a generic alternative ignoring the added value of the digital component (e.g. a usage monitor and connected app supporting adherence) [24^▪^].

## SOCIETAL IMPLICATIONS AND OVER-ARCHING PRINCIPLES

Widespread implementation of digital home monitoring and remote care represents a fundamental change in how healthcare is delivered with significant societal implications including digital inequities, quality of communication and data management and security.

### Digital inequities

Meeting the principles of ‘fairness’ in digital healthcare provision [90], means ensuring equitable access to technology [91]. Without safeguards, implementation of digital healthcare will exclude the most marginalized whether for reasons of age/gender, geography, deprivation or lack of technology skills [25]. At a policy level, this not only means ensuring the technology infrastructure for reliable internet and mobile coverage but also resolving the current lack of clarity over funding streams and reimbursement for digital healthcare. In low-income and middle-income countries, unstable electricity supplies further compromise access [63^▪^], and gender imbalance in mobile ownership may disadvantage women [92]. Other barriers are lack of familiarity with technology, especially in the elderly and language barriers in ethnic minority groups [93].

The e-health literacy framework identifies seven domains for understanding capacity to use and benefit from health technology and provides a potential checklist for the development and improvement of e-health services [94]. Well resourced training programmes and on-going support will be an essential implementation strategy to ensure that patients, professionals and organisations are able optimally to use the system. Appropriately designed interfaces that make no assumptions about prior technology knowledge are acceptable to COPD populations (often elderly, deprived and with multimorbidity) [95,96]. As such, engaging with potentially marginalized groups in the development of digital health initiatives is essential [25].

### Quality of communication in remote digital care

The quality of communication when healthcare professionals and patients are connected digitally rather than face-to-face is an on-going theme in qualitative research [26,97], with both patients and professionals raising concerns about loss of ‘human-ness’ and ‘empathy’ in remote consultations [97,98]. In contrast, digital home monitoring could enhance patients’ confidence in their ability to self-manage, especially if there is a perception of professional oversight [99,100]. In the context of ‘hospital-at-home’; daily remote (video) communication was reported as reassuring [101]. Support for self-management of stable conditions could be flexible [102], and initiated by the patient with the ability to transmit monitoring logs to inform discussions [103]. Remote monitoring and communication have been used as a welcomed adjunct to face-to-face supportive and palliative care [104]. A common observation was that remote care ‘worked better’ when the interaction was building on an existing relationship [105]. Communication skills, such listening, encouraging questioning, emphasizing choice and investing time in developing rapport may help develop relationships in remote consultations [26], and the increased use of video consulting may overcome some of the ‘distance’ problems [104].

### Data management and security

Digital healthcare generates ‘big data’ of considerable value to clinical practice, public health and research, but individuals have concerns about data privacy and security of their electronic health records and – in a connected digital healthcare system – their personal logs [106]. Robust legislation is essential [e.g. General Data Protection Regulation (GDPR) [107]], but patients need to be confident that innovative digital healthcare interventions are implemented to the highest standards. Specific challenges include the potential breach of privacy with the use of visual sensors and videos, supported by artificial intelligence and augmented reality that can be used to check inhaler technique [108–110]. Silhouettes in conjunction with wearable accelerometer devices may reduce the chance of identification [111].

Cloud computing services (Amazon AWS, from Google GCP and Microsoft Azure) provide quick and flexible options for setting up home monitoring services and are widely used in the digital health field. Depending on the privacy and security required, patient data can be stored on the cloud or in the healthcare organizations’ premises/safe haven. Scaling up local digital interventions to multisector, regional, national – or potentially in the future international – healthcare systems, will need a multidisciplinary implementation team with representatives from patients to policy makers, providing expertise in clinical and healthcare management, system architecture, data security and quality management to design and implement a trusted secure system [112].

## CONCLUSION

Despite high-level promotion by WHO, EU, national policies, most healthcare systems are not yet ready to implement large-scale digital home monitoring. Support for organizational change and professional development will be needed to establish and sustain digital healthcare (e.g. practical resources, skills training, proper reimbursement, integration with existing patient management systems, transferability of data across settings, trusted data security). Understanding and addressing the implementation challenges posed by gaps in policy, regulatory, financial and technical infrastructure is essential to support implementation of equitable digital respiratory care that is acceptable to patients and professionals. To advance the vision of connected healthcare will require multinational collaboration involving a broad range of stakeholders to deliver large-scale implementation studies. The European Respiratory Society has just funded a Clinical Research Collaboration ‘CONNECT’ [113], which will develop a global multidisciplinary network and lay the groundwork for major funding applications with the aim of moving forward in the area of digital respiratory care.

## Acknowledgements

*None.*

### Financial support and sponsorship

*None.*

### Conflicts of interest

*H.P. has received speaker fees from Teva and Sandoz outside the submitted work. She holds, or has recently held research grants within the University of Edinburgh from the National Institute for Health and Care Research, Asthma and Lung UK, Innovate UK.*

*J.F.M.v.B. received grants and/or consultancy fees from AstraZeneca, Chiesi, European Commission COST (COST Action 19132 ‘ENABLE’), GSK, Novartis, Pfizer, Teva and Trudell Medical, outside the submitted work and all paid to his institution. C.y.H. is a visitor in the University of Edinburgh and is a senior consultant in digital health at Deloitte. Her research with the University of Edinburgh, is independent from, and not financially supported by Deloitte. Her views in this publication are her own, and not those of the Deloitte. Neither she, nor Deloitte, stand to gain financially from this work.*

## REFERENCES AND RECOMMENDED READING

Papers of particular interest, published within the annual period of review, have been highlighted as:

▪ of special interest
▪▪ of outstanding interest

## References

[1] World Health Organization. Global diffusion of eHealth: making universal health coverage achievable. Report of the third global survey on eHealth. 2019. Available at: https://www.who.int/publications/i/item/9789241511780. [Accessed 15 January 2023]

[2] World Health Organization. Report on the WHO Symposium on the future of Digital Health Systems in the European Region. Available at: https://apps.who.int/iris/bitstream/handle/10665/329032/9789289059992-eng.pdf. [Accessed 15 January 2023]

[3] Aceto G Persico V Pescapé A. The role of information and communication technologies in healthcare: taxonomies, perspectives, and challenges. J Netw Comput Appl 2018; 107:125–154.

[4] Vindrola-Padros C Singh KE Sidhu MS. Remote home monitoring (virtual wards) for confirmed or suspected COVID-19 patients: a rapid systematic review. EClinicalMedicine 2021; 37:100965. 34179736 10.1016/j.eclinm.2021.100965 PMC8219406

[5] World Health Organization. The impact of the COVID-19 pandemic on noncommunicable disease resources and services: results of a rapid assessment. 2020. Available at: https://www.who.int/publications/i/item/9789240010291. [Accessed 15 January 2023]

[6] Ágh T van Boven JFM Wettermark B. for ENABLE Collaborators. A cross-sectional survey on medication management practices for noncommunicable diseases in Europe during the second wave of the COVID-19 pandemic. Front Pharmacol 2021; 12:685696. 34163364 10.3389/fphar.2021.685696 PMC8216671

[7] Duiverman ML Vonk JM Bladder G. Home initiation of chronic noninvasive ventilation in COPD patients with chronic hypercapnic respiratory failure: a randomised controlled trial. Thorax 2020; 75:244–252. 31484786 10.1136/thoraxjnl-2019-213303 PMC7063397

[8] Health Foundation and Nuffield Trust. The remote care revolution during Covid-19. Available at: https://www.nuffieldtrust.org.uk/files/2020-12/QWAS/digital-and-remote-care-in-covid-19.html#1. [Accessed 15 January 2023]

[9] Dierick BJH Been-Buck S Klemmeier T. Digital spacer data driven COPD inhaler adherence education: the OUTERSPACE proof-of-concept study. Respir Med 2022; 201:106940. 35933835 10.1016/j.rmed.2022.106940

[10▪] Chan A De Simoni A Wileman V. Digital interventions to improve adherence to maintenance medication in asthma. Cochrane Database Syst Rev 2022; (6): Art. No.: CD013030. 10.1002/14651858.CD013030.pub2 PMC9188849 35691614

[11] Pinnock H. Connecting professionals and patients: how technology can support asthma self-management. Respir Drug Deliv Europe 2017; 1:43–52.

[12] Davies GA Alsallakh MA Sivakumaran S. Impact of COVID-19 lockdown on emergency asthma admissions and deaths: national interrupted time series analyses for Scotland and Wales. Thorax 2021; 76:867–873. 33782079 10.1136/thoraxjnl-2020-216380

[13] Cox NS Dal Corso S Hansen H. Telerehabilitation for chronic respiratory disease. Cochrane Database Syst Rev 2021; (1): Art. No.: CD013040. 10.1002/14651858.CD013040.pub2 PMC8095032 33511633

[14] Hanlon P Daines L Campbell C. Telehealth interventions to support self-management of long-term conditions: a systematic meta-review of diabetes, heart failure, asthma, chronic obstructive pulmonary disease and cancer. J Med Internet Res 2017; 19:e172. 28526671 10.2196/jmir.6688 PMC5451641

[15] McCabe C McCann M Brady AM. Computer and mobile technology interventions for self-management in chronic obstructive pulmonary disease. Cochrane Database Syst Rev 2017; (5): Art. No.: CD011425. 10.1002/14651858.CD011425.pub2 PMC6481891 28535331

[16] Taylor GMJ Dalili MN Semwal M. Internet-based interventions for smoking cessation. Cochrane Database Syst Rev 2017; (9): Art. No.: CD007078. 10.1002/14651858.CD007078.pub5 PMC6703145 28869775

[17] Whittaker R McRobbie H Bullen C. Mobile phone text messaging and app-based interventions for smoking cessation. Cochrane Database Syst Rev 2019; (10): Art. No.: CD006611. 10.1002/14651858.CD006611.pub5 PMC6804292 31638271

[18] Janjua S Carter D Threapleton CJD. Telehealth interventions: remote monitoring and consultations for people with chronic obstructive pulmonary disease (COPD). Cochrane Database Syst Rev 2021; (7): Art. No.: CD013196. 10.1002/14651858.CD013196.pub2 PMC8543678 34693988

[19▪] Janjua S Bancho E Threapleton CJD. Digital interventions for the management of chronic obstructive pulmonary disease. Cochrane Database Syst Rev 2021; (4): Art. No.: CD013246. 10.1002/14651858.CD013246.pub2 PMC8094214 33871065

[20] Kew KM Cates CJ. Home telemonitoring and remote feedback between clinic visits for asthma. Cochrane Database Syst Rev 2016; (8): Art. No.: CD011714. 10.1002/14651858.CD011714.pub2 PMC7433298 27486836

[21] Pinnock H Barwick M Carpenter C. for the StaRI group. Standards for Reporting Implementation Studies (StaRI) statement. BMJ 2017; 347:f6753. 10.1136/bmj.i6795 PMC5421438 28264797

[22] Pinnock H Epiphaniou E Pearce G. Implementing supported self-management for asthma: a systematic review of implementation studies. BMC Med 2015; 13:127. 26032941 10.1186/s12916-015-0361-0 PMC4465463

[23] Ayaz M Pasha MF Alzahrani MY. The Fast Health Interoperability Resources (FHIR) standard: systematic literature review of implementations, applications, challenges and opportunities. JMIR Med Inform 2021; 9:e21929. 34328424 10.2196/21929 PMC8367140

[24▪] Kardas P Bago M Barnestein-Fonseca P. Reimbursed medication adherence enhancing interventions in 12 European countries: current state of the art and future challenges. Front Pharmacol 2022; 13:944829. 36034792 10.3389/fphar.2022.944829 PMC9403510

[25] Latulippe K Hamel C Giroux D. Social health inequalities and eHealth: a literature review with qualitative synthesis of theoretical and empirical studies. J Med Internet Res 2017; 19:e6731. 10.2196/jmir.6731 PMC5427250 28450271

[26] Miller EA. The technical and interpersonal aspects of telemedicine: effects on doctor–patient communication. J Telemed Telecare 2003; 9:1–7. 12641885 10.1258/135763303321159611

[27] van Horck M Winkens B Wesseling G. Early detection of pulmonary exacerbations in children with cystic fibrosis by electronic home monitoring of symptoms and lung function. Scientific Rep 2017; 7:1–7. 10.1038/s41598-017-10945-3 PMC5617859 28955051

[28] Murphy LA Harrington P Taylor SJ. Clinical-effectiveness of self-management interventions in chronic obstructive pulmonary disease: an overview of reviews. Chron Respir Dis 2017; 14:276–288. 28774200 10.1177/1479972316687208 PMC5720233

[29] Glyde H Blythin A Wilkinson T. Exacerbation predictive modelling using real-world data from the myCOPD app. Eur Respir J 2022; 60:1116. 10.1016/j.heliyon.2024.e31201 PMC11128912 38803869

[30] Exarchos KP Beltsiou M Votti CA Kostikas K. Artificial intelligence techniques in asthma: a systematic review and critical appraisal of the existing literature. Eur Respir J 2020; 56:2000521. 32381498 10.1183/13993003.00521-2020

[31] Barbosa MT Sousa CS Morais-Almeida M. Telemedicine in COPD: an overview by topics. J COPD 2020; 17:601–617. 10.1080/15412555.2020.1815182 32892650

[32] Nakshbandi G Moor C Wijsenbeek M. Home monitoring for patients with ILD and the COVID-19 pandemic. Lancet Respir Med 2020; 8:1172–1174. 33075296 10.1016/S2213-2600(20)30452-5 PMC7567485

[33] Harada T Shibuya Y Kamei T. Effectiveness of telenursing for people with lung cancer at home: a systematic review and meta-analysis. Jpn J Nurs Sci 2022; 20:e12516. 36266923 10.1111/jjns.12516

[34] Feng Y Wang Y Zeng C Mao H. Artificial intelligence and machine learning in chronic airway diseases: focus on asthma and chronic obstructive pulmonary disease. Int J Med Sci 2021; 18:2871. 34220314 10.7150/ijms.58191 PMC8241767

[35] Canny A Donaghy E Murray V. Patient views on asthma diagnosis and how a clinical decision support system could help: a qualitative study. Health Expect 2023; 26:307–317. 36370457 10.1111/hex.13657 PMC9854294

[36] Kouri A Yamada J Lam Shin Cheung J. Do providers use computerized clinical decision support systems? A systematic review and meta-regression of clinical decision support uptake. Implement Sci 2022; 17:1–11. 35272667 10.1186/s13012-022-01199-3 PMC8908582

[37] Tsai C-L Clark S Camargo CA Jr. Risk stratification for hospitalization in acute asthma: the CHOP classification tree. Am J Emerg Med 2010; 28:803–808. 20837258 10.1016/j.ajem.2009.04.009 PMC2939861

[38] Noble M Burden A Stirling S. Predicting asthma-related crisis events using routine electronic healthcare data. Br J Gen Pract 2021; 71x:e948–e957. 10.3399/BJGP.2020.1042 PMC8544121 34133316

[39] Colom R Gonzalez C Herranz JC. Computational modelling for enhanced management of multimorbidity. Eur Respir J 2022; 60:2529.

[40] Patil R Shrivastava R Juvekar S. Specialist to nonspecialist teleconsultations in chronic respiratory disease management: a systematic review. J Glob Health 2021; 11:04019. 34326988 10.7189/jogh.11.04019 PMC8294828

[41] Dixon E Dick K Ollosson S. Telemedicine and cystic fibrosis: do we still need face-to-face clinics? Paediatr Respir Rev 2021; 42:23–28. 34215541 10.1016/j.prrv.2021.05.002

[42] Diedrich L Dockweiler C. Video-based teleconsultations in pharmaceutical care - a systematic review. Res Social Adm Pharm 2021; 17:1523–1531. 33341405 10.1016/j.sapharm.2020.12.002

[43] Ferreira-Cardoso H Jácome C Silva S. Lung auscultation using the smartphone—feasibility study in real-world clinical practice. Sensors 2021; 21:4931. 34300670 10.3390/s21144931 PMC8309818

[44] Sourour A Karray MM Gargouri R. Lung sounds classification with artificial intelligence. Eur Respir J 2022; 60:3473.

[45] Cerdán-de-Las-Heras J Balbino F Løkke A. Effect of a new tele-rehabilitation program versus standard rehabilitation in patients with chronic obstructive pulmonary disease. J Clin Med 2021; 11:11. 35011755 10.3390/jcm11010011 PMC8745243

[46] Calthorpe RJ Smith S Gathercole K Smyth AR. Using digital technology for home monitoring, adherence and self-management in cystic fibrosis: a state-of-the-art review. Thorax 2020; 75:72–77. 31594802 10.1136/thoraxjnl-2019-213233

[47] Holland AE Malaguti C Hoffman M. Home-based or remote exercise testing in chronic respiratory disease, during the COVID-19 pandemic and beyond: a rapid review. Chron Respir Dis 2020; 17:1479973120952418. 32840385 10.1177/1479973120952418 PMC7450293

[48] Rutkowski S. Management challenges in chronic obstructive pulmonary disease in the COVID-19 pandemic: telehealth and virtual reality. J Clin Med 2021; 10:1261. 33803853 10.3390/jcm10061261 PMC8003143

[49] Cerdán de Las Heras J Tulppo M Kiviniemi AM. Augmented reality glasses as a new tele-rehabilitation tool for home use: patients’ perception and expectations. Disability Rehabil Assist Technol 2022; 17:480–486. 10.1080/17483107.2020.1800111 32750254

[50] Shields MD ALQahtani F Rivey MP McElnay JC. Mobile direct observation of therapy (MDOT) - a rapid systematic review and pilot study in children with asthma. PLoS One 2018; 13:e0190031. 29401500 10.1371/journal.pone.0190031 PMC5798760

[51] McCrossan P O’Donoghue D McElnay JC Shields MD. The use of remote video directly observed therapy to improve both inhaler technique and adherence to asthma medications. Front Public Health 2022; 10:965629. 36276358 10.3389/fpubh.2022.965629 PMC9581185

[52] Tony SM Abdelrahman MA Abd Elsalam M. Effect of using acoustic flo-tone training device and its smartphone application on enhancing inhalation technique from metered-dose inhaler with spacer in asthmatic children. Exp Lung Res 2022; 48:224–238. 35997099 10.1080/01902148.2022.2113573

[53] Häussermann S Andersen L Frisch M. Adherence to inhaled drugs–more than just reminders and nudging. Eur Respir J 2022; 60:4697.

[54] Dhruve H Jackson DJ. Assessing adherence to inhaled therapies in asthma and the emergence of electronic monitoring devices. Eur Respir Rev 2022; 31:210271. 35613744 10.1183/16000617.0271-2021 PMC9488666

[55] O’Connor A Tai A Brinn M. The acceptability of using augmented reality as a mechanism to engage children in asthma inhaler technique training: qualitative interview study with deductive thematic analysis. JMIR Pediatr Parent 2023; 6:e40231. 36637889 10.2196/40231 PMC9883739

[56] Shah A Hussain-Shamsy N Strudwick G. Digital health interventions for depression and anxiety among people with chronic conditions: scoping review. J Med Internet Res 2022; 24:38030. 10.2196/38030 PMC9555324 36155409

[57] Torous J Bucci S Bell IH. The growing field of digital psychiatry: current evidence and the future of apps, social media, chatbots, and virtual reality. World Psych 2021; 20:318–335. 10.1002/wps.20883 PMC8429349 34505369

[58] Andreou A Dhand A Vassilev I. Understanding online and offline social networks in illness management of older patients with asthma and chronic obstructive pulmonary disease: mixed methods study using quantitative social network assessment and qualitative analysis. JMIR Form Res 2022; 6:35244. 10.2196/35244 PMC9157321 35579933

[59] Asthma UK. Indoor asthma triggers. Available at: https://www.asthma.org.uk/advice/triggers/indoor-environment/. [Accessed 1 February 2023]

[60] Venkataramanan R Thirunarayan K Jaimini U. Determination of personalized asthma triggers from multimodal sensing and a mobile app: observational study. JMIR Pediatr Parent 2019; 2:e14300. 31518318 10.2196/14300 PMC6716491

[61] Bui AA Hosseini A Rocchio R. Biomedical real-time health evaluation (BREATHE): toward an mHealth informatics platform. JAMIA Open 2020; 3:190–200. 32734159 10.1093/jamiaopen/ooaa011 PMC7382637

[62] Hui CY McKinstry B Mclean S. Assessing the technical feasibility of a flexible, integrated Internet-of-things connected for asthma (C4A) system to support self-management: a mixed method study exploring patients’ and healthcare professionals’ perspectives. JAMIA Open 2022; 5:ooac110. 36601366 10.1093/jamiaopen/ooac110 PMC9801970

[63▪] Hui CY Abdullah A Ahmed Z. Mapping national information and communication technology (ICT) infrastructure to the requirements of potential digital health interventions in low- and middle-income countries. J Glob Health 2022; 2:04094. 10.7189/jogh.12.04094 PMC9804211 36579436

[64] Orchard P Agakova A Pinnock H. Improving prediction of risk of hospital admission in chronic obstructive pulmonary disease: application of machine learning to telemonitoring data. J Med Internet Res 2018; 20:e263. 30249589 10.2196/jmir.9227 PMC6231768

[65] Tsang KCH Pinnock H Wilson AM. Mobile device monitoring to inform prediction of asthma attacks: an observational study AAMOS-00. BMJ Open 2022; 12:e064166. 10.1136/bmjopen-2022-064166 PMC9535155 36192103

[66] Hui CY McKinstry B Fulton O. Patients’ and clinicians’ visions of a future internet-of-things system to support asthma self-management: mixed methods study. J Med Internet Res 2021; 23:e22432. 33847592 10.2196/22432 PMC8080146

[67] Eikholt AA Wiertz MB Hew M. Electronic monitoring devices to support inhalation technique in patients with asthma: a narrative review. Curr Treat Options Allergy 2023; 10:28–52.

[68▪] Wijsenbeek MS Moor CC Johannson KA. Home monitoring in interstitial lung diseases. Lancet Respir Med 2023; 11:97–110. 36206780 10.1016/S2213-2600(22)00228-4

[69] Gerke S Shachar C Chai PR Cohen IG. Regulatory, safety, and privacy concerns of home monitoring technologies during COVID-19. Nature Med 2020; 26:1176–1178. 32770164 10.1038/s41591-020-0994-1 PMC7435203

[70] Johnson KB Wei WQ Weeraratne D. Precision medicine, AI, and the future of personalized healthcare. Clin Translational Sci 2021; 14:86–93. 10.1111/cts.12884 PMC7877825 32961010

[71] Khanijahani A Iezadi S Dudley S. Organizational, professional, and patient characteristics associated with artificial intelligence adoption in healthcare: a systematic review. Health Policy Technol 2022; 11:100602.

[72] O’Brien N, Van Dael J, Clarke J, *et al.* Addressing racial and ethnic inequities in data-driven health technologies. Available at: https://spiral.imperial.ac.uk/bitstream/10044/1/94902/2/Imperial_IGHI_AddressingRacialandEthnicInequities%20_Report.pdf. [Accessed 30 March 2023]

[73] Abdolkhani R Gray K Borda A. Patient-generated health data management and quality challenges in remote patient monitoring. JAMIA Open 2019; 2:471–478. 32025644 10.1093/jamiaopen/ooz036 PMC6993998

[74] Fan KG Mandel J Agnihotri P. Remote patient monitoring technologies for predicting chronic obstructive pulmonary disease exacerbations: review and comparison. JMIR mHealth uHealth 2020; 8:e16147. 32348262 10.2196/16147 PMC7273236

[75] Quach S Benoit A Oliveira A. Features and quality of COPD self-management apps in the Android marketplace. Eur Respir J 2022; 60: (Suppl 66): 574.

[76] Ghimire S Iskandar D van der Borg-Boekhout R. Combining digital adherence technology and therapeutic drug monitoring for personalised tuberculosis care. Eur Respir J 2022; 60:2201690. 10.1183/13993003.01690-2022 36356974

[77] Carpenter DM Jurdi R Roberts CA. A review of portable electronic spirometers: implications for asthma self-management. Curr Allergy Asthma Rep 2018; 18:53. 30145683 10.1007/s11882-018-0809-3

[78] UK Department for Business, Energy & Industrial Strategy. CE marking: how a product complies with EU safety, health and environmental requirements, and how to place a CE marking on your product (Updated 2022). Available at: https://www.gov.uk/guidance/ce-marking#overview. [Accessed 15 January 2023]

[79] US Food and Drug Administration. Medical devices. Available at: https://www.fda.gov/medical-devices. [Accessed 15 January 2023]

[80] UK Department for Business, Energy & Industrial Strategy. Using the UKCA marking (updated 2022). Available at: https://www.gov.uk/guidance/using-the-ukca-marking. [Accessed 15 January 2023]

[81] Fraser AG Butchart EG Szymański P. The need for transparency of clinical evidence for medical devices in Europe. Lancet 2018; 392:521–530. 30017550 10.1016/S0140-6736(18)31270-4

[82] Organisation for the Review of Care and Health Apps (ORCHA). Find the Best Health Apps. Available at: https://appfinder.orcha.co.uk/Date. [Accessed 15 January 2023]

[83] Hoyte FCL Mosnaim GS Rogers L. Effectiveness of a digital inhaler system for patients with asthma: a 12-week, open-label, randomized study (CONNECT1). J Allergy Clin Immunol Pract 2022; 10:2579–2587. 36038131 10.1016/j.jaip.2022.08.023

[84▪] MacHale E, Greene G, Mulvey C, *et al*. Use of digital measurement of medication adherence and lung function to guide the management of uncontrolled asthma: the INCA Sun randomized clinical trial. Lancet Respir Med. Available online 21 March 2023. 10.1016/S2213-2600(22)00534-3. 36963417

[85] International Medical Device Regulators Forum, AIMD working group. Artificial Intelligence Medical Devices. Available at: https://www.imdrf.org/working-groups/artificial-intelligence-medical-devices. [Accessed 6 February 2023]

[86] Food and Drug Administration. Digital Health Technologies for Remote Data Acquisition in Clinical Investigations: Draft Guidance for Industry, Investigators, and Other Stakeholders. January 2022. Available at: https://www.fda.gov/regulatory-information/search-fda-guidance-documents/digital-health-technologies-remote-data-acquisition-clinical-investigations. [Accessed 6 January 2023]

[87▪] Rakers MM van Os HJ Recourt K. Perceived barriers and facilitators of structural reimbursement for remote patient monitoring, an exploratory qualitative study. Health Policy Technol 2023; 12:100718.

[88] van Boven JFM Cushen B Sulaiman I. Personalising adherence-enhancing interventions using a smart inhaler in patients with COPD: an exploratory cost-effectiveness analysis. NPJ Prim Care Respir Med 2018; 28:24. 29950601 10.1038/s41533-018-0092-8 PMC6021429

[89] van Boven JFM van de Hei SJ Sadatsafavi M. Making sense of cost-effectiveness analyses in respiratory medicine: a practical guide for nonhealth economists. Eur Respir J 2019; 53:1801816. 30578398 10.1183/13993003.01816-2018

[90] UK Government Digital Service. Data Ethics Framework 2020. Available at: https://www.gov.uk/government/publications/data-ethics-framework. [Accessed 15 January 2023]

[91] Hernandez K, Roberts T. Leaving no one behind in a digital world. K4D Emerging Issues Report. Brighton, UK: Institute of Development Studies.

[92] Global System for Mobile Communications. Connected Women The Mobile Gender Gap Report 2021. Available at: https://www.gsma.com/r/wp-content/uploads/2021/06/The-Mobile-Gender-Gap-Report-2021.pdf. [Accessed 15 January 2023]

[93] Sinha S Kern LM Gingras LF. Implementation of video visits during COVID-19: lessons learned from a primary care practice in New York City. Front Public Health 2020; 8:514. 33042950 10.3389/fpubh.2020.00514 PMC7527590

[94] Norgaard O Furstrand D Klokker L. The e-health literacy framework: a conceptual framework for characterizing e-health users and their interaction with e-health systems. Knowl Manag E-Learn 2015; 7:522–540.

[95] Pinnock H Hanley J McCloughan L. Effectiveness of telemonitoring integrated into existing clinical services on hospital admission for exacerbation of chronic obstructive pulmonary disease: researcher blind, multicentre, randomised controlled trial. BMJ 2013; 347:f6070. 24136634 10.1136/bmj.f6070 PMC3805483

[96] Jakobsen AS Laursen LC Rydahl-Hansen S. Home-based telehealth hospitalization for exacerbation of chronic obstructive pulmonary disease: findings from ‘the virtual hospital’ trial. Telemed J E Health 2015; 21:364–373. 25654366 10.1089/tmj.2014.0098 PMC4432494

[97] Wanat M Hoste ME Gobat NH. Patients’ and clinicians’ perspectives on the primary care consultations for acute respiratory infections during the first wave of the COVID-19 pandemic: an eight-country qualitative study in Europe. BJGP Open 2022; 6:0172. 10.3399/BJGPO.2021.0172 PMC9447319 35031559

[98] Boers SN Jongsma KR Lucivero F. Part 2: clinical implementation of eHealth in Primary Care: addressing the ethical dimensions. Eur J Gen Pract 2020; 26:26–32. 31663394 10.1080/13814788.2019.1678958 PMC7034078

[99] Pinnock H Slack R Pagliari C. Understanding the potential role of mobile phone based monitoring on asthma self-management: qualitative study. Clin Exp Allergy 2007; 3:794–802. 10.1111/j.1365-2222.2007.02708.x 17456228

[100] Ure J Hanley J Pinnock H. Piloting tele-monitoring in chronic obstructive pulmonary disease: a mixed methods exploration of issues in design and implementation. Prim Care Respir J 2012; 21:57–64. 21785816 10.4104/pcrj.2011.00065 PMC6548305

[101] Jakobsen AS Laursen LC Rydahl-Hansen S. Home-based telehealth hospitalization for exacerbation of chronic obstructive pulmonary disease: findings from ‘the virtual hospital’ trial. Telemed E-Health 2015; 21:364–373. 10.1089/tmj.2014.0098 PMC4432494 25654366

[102] Kielmann T Huby G Powell A. From support to boundary: a qualitative study of the border between self care and professional care. Pat Ed Counsel 2010; 79:55–61. 10.1016/j.pec.2009.07.015 19709844

[103] MacNab M Lee SH McCloughan L. Oximetry-supported self-management for chronic obstructive pulmonary disease: mixed method evaluation of a pilot project. BMC Health Serv Res 2015; 15:485. 26503028 10.1186/s12913-015-1135-2 PMC4624181

[104] Funderskov KF Boe Danbjørg D Jess M. Telemedicine in specialised palliative care: healthcare professionals’ and their perspectives on video consultations - a qualitative study. J Clin Nurse 2019; 28:3966–3976. 10.1111/jocn.15004 31328336

[105] Orlando JF Beard M Kumar S. Systematic review of patient and caregivers’ satisfaction with telehealth videoconferencing as a mode of service delivery in managing patients’ health. PloS One 2019; 14:e0221848. 31469865 10.1371/journal.pone.0221848 PMC6716655

[106] European Lung Foundation. Patient Organisation Networking Day 2021. Available at: https://europeanlung.org/en/elf-patient-organisation-networking-day-2021/. [Accessed 15 January 2023]

[107] GDPR.eu. Complete guide to GDPR compliance. Available at: https://gdpr.eu. [Accessed 15 January 2023]

[108] Hui CY McKinstry B Fulton O. Patients’ and clinicians’ perceived trust in internet-of-things systems to support asthma self-management: qualitative interview study. JMIR mHealth uHealth 2021; 9:e24127. 34269684 10.2196/24127 PMC8325078

[109] Tay TR van Boven JFM Chan A. Electronic inhaler monitoring for chronic airway disease: development and application of a multidimensional efficacy framework. J Allergy Clin Immunol Pract 2022; 10:1189–1201. 34915225 10.1016/j.jaip.2021.11.027

[110] Zhao M Hoti K Wang H. Assessment of medication self-administration using artificial intelligence. Nat Med 2021; 27:727–735. 33737750 10.1038/s41591-021-01273-1

[111] Masullo A Burghardt T Damen D. Person re-id by fusion of video silhouettes and wearable signals for home monitoring applications. Sensors 2020; 20:2576. 32369960 10.3390/s20092576 PMC7248699

[112] Digital Implementation Investment Guide (DIIG): Integrating Digital Interventions into Health Programmes. World Health Organisation. 15 September 2022. Available at: https://www.who.int/publications/i/item/9789240010567. [Accessed 6 February 2023]

[113] European Respiratory Society. Ongoing Clinical Research Collaborations. Available at: https://www.ersnet.org/science-and-research/ongoing-clinical-research-collaborations/. [Accessed 30 March 2023]

